# An Exploratory Study of Laser Scribing Quality through Cross-Section Scribing Profiles

**DOI:** 10.3390/mi14112020

**Published:** 2023-10-30

**Authors:** Ruqi Chen, Shing Chang, Shuting Lei

**Affiliations:** Department of Industrial and Manufacturing Systems Engineering, Kansas State University, Manhattan, KS 66506, USA; chenruqi@ksu.edu

**Keywords:** laser scribing, aluminum thin film on silicon, target area ratio (RA), mean square error (MSE)

## Abstract

This article presents a novel approach for evaluating laser scribing quality through cross-section profiles generated from a three-dimensional optical profiler. Existing methods for assessing scribing quality only consider the width and depth of a scribe profile. The proposed method uses a cubic spline model for cross-section profiles. Two quality characteristics are proposed to assess scribing accuracy and consistency. Accuracy is measured by the ratio of the actual laser-scribed area to the target area (RA), which reflects the deviation from the desired profile. The mean square error (MSE) is a measure of how close each scribed cross-section under the same scribing conditions is to the fitted cubic spline model. Over 1370 cross-section profiles were generated under 171 scribing conditions. Two response surface polynomial models for RA and MSE were built with 18 scribing conditions with acceptable scribing depth and RA values. Both RA and MSE were considered simultaneously via contour plots. A scatter plot of RA and MSE was then used for Pareto optimization. It was found that the cross-sectional profile of a laser scribe could be accurately represented by a cubic spline model. A multivariate nonlinear regression model for RA and MSE identified pulse energy and repetition rate as the two dominant laser parameters. A Pareto optimization analysis further established a Pareto front, where the best compromised solution could be found.

## 1. Introduction

Laser scribing is a versatile laser micromachining technique that finds applications across various industries, including medical device production [1], automotive parts manufacturing [2], semiconductor processing [3], and solar cell fabrication [4]. It facilitates the creation of shallow scribe lines on surfaces, with exceptional precision in both depth and lateral dimensions [5,6]. Laser scribing presents numerous advantages by enabling the fabrication of high-quality scribe lines and plays a pivotal role in achieving intricate and precise microscale structures across various industries [7]. Moreover, in the domain of solar cell fabrication [8,9,10], laser scribing assumes a critical role in enhancing the efficiency and performance of solar cells.

To ensure the high-quality scribe lines demanded by these applications, researchers have extensively studied the suitability of short pulse lasers, such as picosecond and nanosecond lasers, in laser scribing processes [11,12]. These lasers offer high precision and control, enabling researchers to explore the relationship between pulse energy, pulse duration, and scribing outcomes [13,14]. The short pulse duration allows for rapid energy deposition on the material surface, induced localized heating, and minimized heat diffusion to the surrounding areas. This characteristic is particularly advantageous in laser scribing as it enables precise control over the ablation process while minimizing thermal effects and collateral damage to adjacent regions of the solar cell [15,16,17].

However, the quest for higher productivity and faster processing speeds must be balanced with the need to maintain high scribe quality. Despite the above-mentioned advantages of short pulse lasers, several studies have highlighted potential negative effects, including tapered cross-sectional profiles, thermal damage, microcrack formation, and recast material accumulation [18,19,20]. These issues arise due to the Gaussian beam profile and intense heat generated during the laser scribing process [21,22]. Furthermore, laser stability and variations in surface morphology and composition pose additional challenges, affecting laser–material interaction and resulting in inconsistent scribe quality [23,24]. Hence, optimizing process parameters for different materials and applications is essential.

For process optimization, measures of scribe quality need to be defined. From a geometric point of view, scribe width and depth are often used to measure scribe quality [25,26]. However, width and depth are only two quality characteristics, while a cross-sectional profile provides more complete information on scribing quality. In our pursuit of deeper analysis and heightened comprehension, we turned to the mathematical technique of cubic spline interpolation. This approach is instrumental in estimating values between known data points. By constructing a seamless curve using a series of cubic polynomials, this method ensures both continuity and smoothness. The term ‘spline’ is derived from the flexibility of the curve to adapt to the shape of the data, resembling a flexible strip or spline of wood used by draftsmen. This technique provides a superior means of dissecting scribing profiles, affording us intricate understandings of the interplay between the laser scribing tool and the material, particularly in situations where precision and accuracy hold paramount importance. This methodology proves essential, enabling us to capture even the subtlest nuances and intricate details.

In this study, we present an exploratory investigation of laser scribing quality assessment through cross-sectional scribing profiles. We demonstrate how a cubic spline model allows for interpolation between data points and the fitting of a smooth curve to identify the optimal parameter settings for achieving the desired quality characteristics. Furthermore, we define two measurement statistics for measuring accuracy and consistency and construct a regression model for each statistic to address the parameter optimization process. The proposed methodology aims to identify the optimal laser scribing conditions and offer valuable insights for applications that rely on precise and controlled scribing techniques.

## 2. Experimental Plan and Data Collection

### 2.1. Experimental Plan

**Figure 1:**
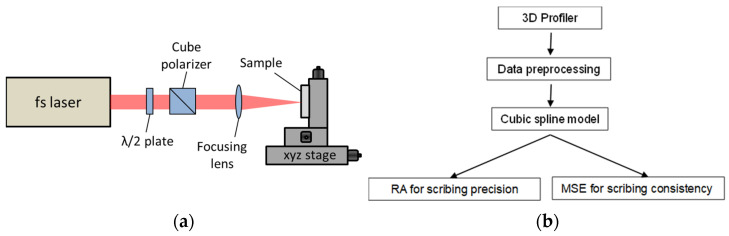
(**a**) Configuration of laser scribing experiment and (**b**) outline of the envisaged data analysis approach.

The laser scribing experimental setup, as depicted in Figure 1a, is outlined in the following bullet points.

Experimental Setup:
  - Laser Scribing Setup:
    - Utilizes a femtosecond (fs) laser.
    - Adjusts pulse energy using a half-wave plate and cube polarizer.
    - Focuses pulse energy to a focal spot on the sample surface with a focusing lens.
Sample Characteristics:
  - Consists of a 300 nm thick aluminum film coated on silicon.
  - Sample position controlled by an XYZ translation stage.
  - Writing direction perpendicular to the beam propagation axis.
Laser Scribing Process:
  - Conducted on the Al-coated silicon sample.
  - Variables Adjusted:
    - Pulse energy (range: 12–360 μJ)
    - Pulse duration (range: 0.184–10 ps)
    - Pulse frequency (range: 250–200,000 Hz)

Over 171 test conditions were implemented, each with three repetitions. The pulse energy density ranged from 4.8 to 240 J/cm^2^.

Post-Scribing Analysis:
  - Examination using a 3D optical profiler.
  - Measure cross-sectional profiles for each scribe.
  - Conducted at a minimum of three distinct locations.

### 2.2. Data Collection and Preprocessing

Following the laser scribing process, the sample underwent scrutiny using a 3D optical profiler, where the cross-sectional profile for each scribe was assessed at a minimum of three distinct locations. The demographic data were subsequently extracted from the measurements and then exported for further comprehensive data analysis.

The data analysis methodology, illustrated in Figure 1b, began with the acquisition of raw data from the 3D optical profiler. Subsequently, a data preprocessing phase was implemented to effectively eliminate any noise or outliers, thereby ensuring the integrity of the dataset. Following this, a comprehensive characterization of each scribe’s cross-sectional profile was conducted, encompassing crucial parameters such as depth, width, and overall shape. This critical step formed the cornerstone for evaluating the quality of the scribing process. The subsequent stage involved the application of a cubic spline to the profiles, which is described in detail in the next section.

## 3. Cubic Spline Interpolation Approach

**Figure 2:**
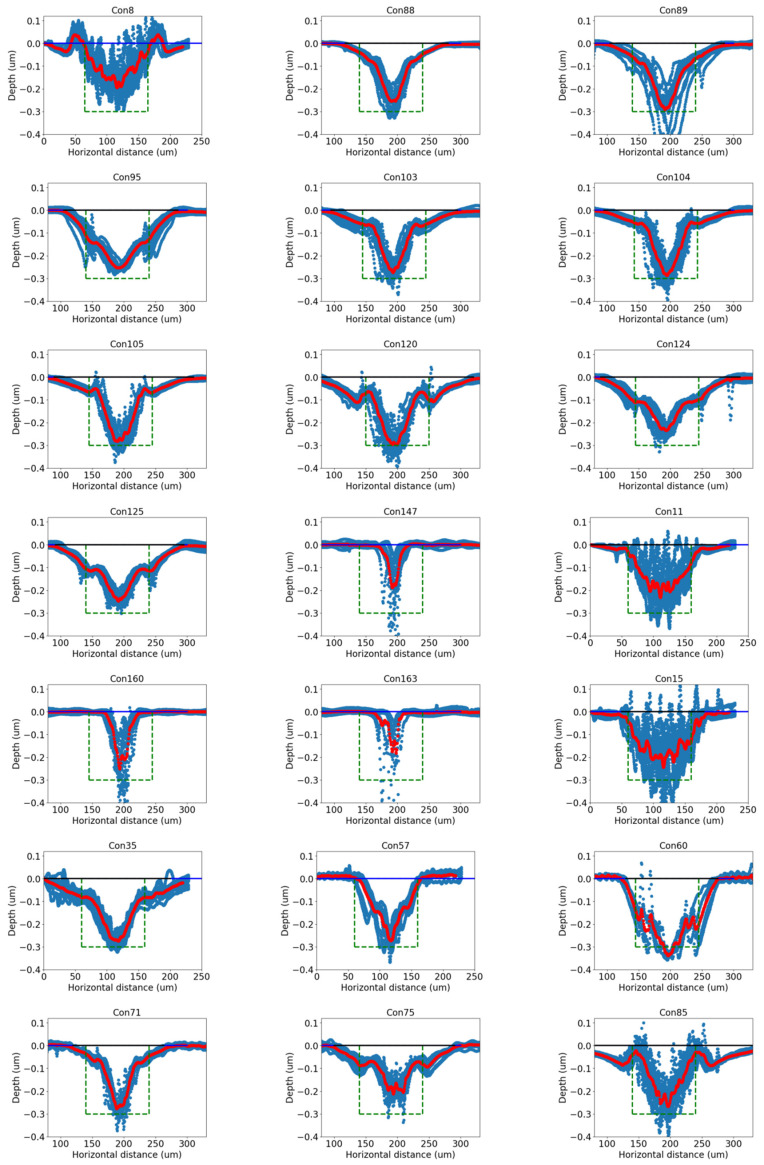
Cubic spline fitting for laser scribing cross-section profiles across a chosen set of 21 scribing conditions. The blue lines in each plot are cross-section profiles from multiple measurements and the red line is the cubic spline fit based on the blue lines.

Examples of spatial series of scribing depths representing scribing profiles are shown in one of the two-dimensional scatter plots in Figure 2. For example, several profiles represented in blue dots can be observed in machine condition 89 (front row and last plot). The shape of profiles provides much more information about scribing quality than mere width and depth alone since the profile shape is much more complex than linear regression models can handle. We chose a cubic spline approach for modeling this application. Once the average cross profiles were computed, both proposed quality characteristics, mean square error (MSE) and the area ratio (RA), could be estimated. The concept and computation of both RA and MSE will be discussed in Section 4.1–Section 4.3. The use of both RA and MSE for process optimization will also be explored in Section 4.4.

**Figure 3:**
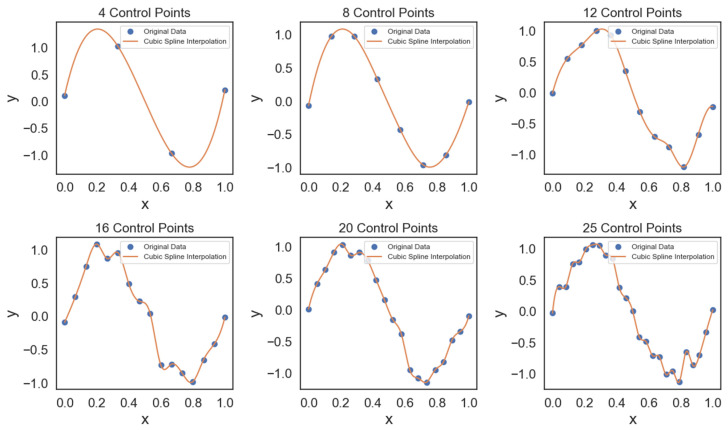
Cubic spline interpolations created using a varying number of control points, from 4 to 25 points.

Cubic spline interpolation is a mathematical technique used to create smooth curves through a series of data points. It works by breaking a dataset into smaller segments and fitting cubic polynomial functions to each segment. This process results in a continuous curve that closely follows the original data, providing a high level of accuracy. As shown in Figure 3, control points are crucial in determining the accuracy of the interpolated curve. Starting with just four control points, the interpolation may exhibit slight variations from the original data. However, as we increase the number of control points to eight, twelve, sixteen, twenty, and finally twenty-five, the cubic spline interpolation becomes progressively refined. This refinement allows it to capture finer details with greater precision. This demonstration highlights how the choice of control points significantly influences the geometric shape of the interpolation, emphasizing the importance of careful parameter selection in achieving the desired outcomes.

The cubic spline method [27] was utilized to fit the laser-scribing datasets. Spline interpolation offers a higher degree of polynomial interpolation while maintaining stability, thanks to Runge’s phenomenon [28]. Cubic spline interpolation is a mathematical technique for constructing a smooth and continuous function that passes through a set of given data points. The method involves dividing the range of the data points into a series of intervals and constructing a cubic polynomial function for each interval. The polynomials are carefully chosen to match the values of the function at the endpoints of each interval while maintaining continuous first and second derivatives [29,30].

Let (*x*_0_, *y*_0_), (*x*_1_, *y*_1_), …, (*x_n_*, *y_n_*) be the given data points, where $x_{i}<x_{i+1}$ or *i* = 0, 1, …, *n −* 1. Our objective is to find a function *S*(*x*) that passes through all the data points and possesses continuous first and second derivatives [31]. To achieve this, we first define a set of cubic polynomials *S_i_*(*x*) on each interval [$x_{i}$, $x_{i+1}$]

(1) $${S_{i}(x)=a}_{i}+b_{i}\left(x-x_{i}\right)+c_{i}{\left(x-x_{i}\right)}^{2}+d_{i}{\left(x-x_{i}\right)}^{3}$$

where $a_{i}$ $a_{i}=y_{i}$, $b_{i}$, $c_{i}$, and $d_{i}$ are constants to be determined for each interval. We can determine these constants by imposing the following conditions on each interval *i* = 0, 1, …, *n* − 1:

(2) $$S_{i}(x_{i})=y_{i}\;\mathrm{for}\;i\;=\;0,\;1,\;\ldots ,\;n\;-\;1.$$

to ensure that each polynomial passes through its corresponding endpoint of the interval;

(3) $${S_{i+1}(x_{i+1})=S}_{i}{(x}_{i+1})\;\mathrm{for}\;i\;=\;0,\;1,\;\ldots ,\;n\;-\;2.$$

to ensure that the polynomials are continuous at the interval endpoints;

(4) $$S_{i+1}^{\prime }\left(x_{i+1}\right)=S_{i}^{\prime }(x_{i+1})\;\mathrm{for}\;i\;=\;0,\;1,\;\ldots ,\;n\;-\;2.$$

to ensure that the first derivatives of the polynomials are continuous at the interval endpoints; and

(5) $$S_{i+1}^{\prime \prime }\left(x_{i+1}\right)=S_{\prime \prime }^{i}(x_{i+1})\;\mathrm{for}\;i\;=\;0,\;1,\;\ldots ,\;n\;-\;2.$$

The second derivatives of the polynomials are continuous at the interval endpoints. Note that we have *n* + 1 data points and *n* intervals, so we have n unknowns (the coefficients $b_{i}$, $c_{i}$, and $d_{i}$ for each interval) and *n* equations (the four conditions above for each interval). To obtain a unique solution, we also need to specify additional boundary conditions. The most common boundary conditions are determined as follows: the second derivatives at the endpoints are set to zero and clamped where the first derivatives at the endpoints are set to given values. Specifically, the natural boundary conditions are *S*″($x_{0}$) = S″($x_{n}$) = 0 and the clamped boundary conditions are S′($x_{0}$) = $m_{0}$ and S′($x_{n}$) = *m_n_*, where $m_{0}$ and *m_n_* are given values. Once we solve the coefficients of each polynomial, we can use the resulting spline function *S*(*x*) to approximate the original function at any point within the range of the data. The formula for the spline function on an interval [$x_{i}$, $x_{i+1}$] is as follows:

(6) $$S(x)\;=\;S_{i}(x),\;\mathrm{for}\;x_{i}\leq x\leq x_{i+1}.$$

Additional boundary conditions can be specified to obtain a unique solution. In cubic spline methodology, the B-spline function is often used to reduce the dimension of the model function while still capturing the key features that define the shape of the profiles. Splines are composed of smoothly connected polynomial segments, with the connection points referred to as knots. The knots do not have to be evenly spaced, and when each segment of a spline is a polynomial of degree d, the spline is referred to as a degree d spline. In our case shown in Equation (1), the cubic spline has a degree 3. In this study, the natural boundary of the cubic spline models was determined by the end points of the depth profile datasets. This property provided a smooth transition beyond the scribed section of a cross-section profile without the need for manual intervention.

## 4. Scribing Quality Evaluation Using Cubic Spline Trajectory

The proposed methodology for evaluating the quality of laser scribing profiles is illustrated as follows. The first step was to collect the cross-sectional profile data for each laser scribe. From the data, the depth of the scribe was calculated, and scribing conditions that met the depth criteria of 250–350 nm were identified. To assess the quality of the scribing profiles, we considered not only the width and depth but also the cross-section profiles. The cross-section profiles were modeled using cubic spline regression, which allowed for a more accurate representation of the profile. Unknown coefficients of a regression model were estimated from multiple profiles under the same scribing condition. Two quality characteristics were introduced to evaluate the scribe quality: the ratio of the actual laser scribed to the target area (RA) and the mean square error (MSE) of the cubic spline model. RA measured the deviation of the estimated profiles (i.e., the fitted cubic spline model) from the desired profile (i.e., a square). MSE evaluated the consistency of a scribe condition by computing the mean square error of each scribe cross-section from the fitted cubic spline model.

To establish correlations between the laser parameters and the dual responses RA and MSE, two response surface models were constructed. The laser parameters included pulse energy (E), pulse duration (tp), and pulse frequency (fp). Based on the fitted models, contour plots could be used to identify possible optimization regions that maximize RA and minimize MSE. Note that a contour plot only contains two laser parameters at a time. We chose the two most significant factors while fixing the least significant factor. This comprehensive workflow offers a reliable method for evaluating laser scribing quality and can be applied in future applications. By considering cross-sectional profiles and using the cubic spline regression, the proposed methodology provides a more accurate and comprehensive approach to assessing laser scribing quality.

### 4.1. Cubic Spline Fit of Laser Scribing Profiles

A cubic spline model was utilized to fit the laser-scribing cross-section profiles in this study. This model ensures a smooth and continuous curve that accurately represents the cross-section data points. Precise representation of a laser scribing cross-section profile is vital for evaluating scribing quality as it allows for a detailed analysis of the scribing process and its outcomes. The flexibility of cubic spline fitting is particularly advantageous in this context as it allows for adjusting the degree of smoothing to achieve the best fit for the given data. Cubic spline fitting has been widely recognized for its effectiveness in handling noisy and irregular data while maintaining the desired smoothness and continuity. This cubic spline property enables laser scribing cross-section profile modeling, which can often exhibit fluctuations and irregularities due to various factors such as material properties, processing parameters, and experimental conditions. The application of cubic spline fitting in this study ensured the accurate representation of laser-scribing cross-section profiles and enabled a comprehensive analysis of the scribing quality under different conditions.

A selected 21 among 171 conditions in Figure 2 were chosen to filter out defective samples via the targeted scribing depth, which measured approximately 300 nm. Specifically, a scribing condition was chosen when the average scribing depth of all samples under this condition fell within a tolerance range (250–350 nm). Figure 2 shows 21 fitted cubic spline cross-section profiles in red. Specifically, 10 profiles were generated for each scribing condition. At each spatial location, 10 depths were averaged. The collection of all averaged depth data was then fed into the proposed cubic spline model to generate the red profiles in Figure 2. Each plot is under one scribing condition. Multiple scribing cross sections shown in blue profiles were used to fit a cubic spline model. These scribed conditions were chosen based on the average scribe depth.

Note that there were large variations in the cross-sectional profiles from sample to sample, even under the same scribing conditions. The main cause of this is believed to be the large differences associated with the deposited laser energy profile, i.e., non-uniform laser intensity and non-uniform energy density over the writing area. Other sources may include plasma/plume shielding of the laser beam, optical property change during laser irradiation, material ejection and redeposition dynamics, etc.

### 4.2. Area Ratio for Scribing Precision Measurement

The scribing precision was assessed through the area ratio, which was defined as the ratio of the laser scribe’s cross-sectional area to the designed target area, with a depth of 300 nm and a width of 100 μm. The target area was obtained by multiplying the width and depth, resulting in a value of 30 μm^2^. The target area is illustrated in Figure 2 as a green box. The actual scribing area for each condition was calculated using the profile data, and a penalty (A_penalty_, the area outside the box) was imposed when the area exceeded the designed area to ensure the accuracy of the measurement. The scribing precision was evaluated using the area ratio (RA), which was computed as the ratio of the actual scribing area (A_actual_) to the target area (A_target_). The equation for calculating the area ratio is as follows:

RA = (A_actual_ − A_penalty_)/A_target_ (7)

In this equation, A_penalty_ represents the area that exceeds the intended target area. When the laser scribing area extended beyond the boundaries of the target, a penalty function was applied to eliminate the portion outside the target box. The penalty function was defined as the integral of the exceeded trace, which quantified the area where the laser scribing extended beyond the intended target boundaries. It measured the extent of the exceeded area by integrating the exceeded trace. By subtracting the penalty value (A_penalty_) from the actual scribing area (A_actual_), the area ratio calculation ensured that only the scribing area within the target boundaries was considered. A_actual_ is the area of the scribe profiles falling within the target area, which was a square (i.e., the green box in Figure 2). Dividing the adjusted scribing area by the target area (A_target_) provided a normalized measure of the scribing process’s adherence to the desired specifications.

In Equation (7), a large area ratio (RA) indicates better precision in achieving the targeted scribe area. The actual scribe area closely matching the targeted value and achieving a maximum RA (rectangularity) of 1 may not be feasible in practice due to the inherent tapering of the laser beam shape, which differs from the ideal rectangular shape. The scribing process was highly accurate and achieved the desired outcome with minimal deviation. For the limits of the equation, there may be several specific factors that affect practical application. The first is material characteristics: certain materials may inherently limit the achievable RA due to their properties. For instance, brittle or heat-sensitive materials may introduce constraints on precision. The second is equipment precision: the level of precision achievable is also influenced by the capabilities of the scribing equipment. Advanced machinery may enable higher Ras, but there may be diminishing returns beyond a certain point. While a large RA generally signifies better precision, its practical application should be assessed considering material properties, equipment capabilities, and the specific demands of the application. In summary, the target RA value is a function of these aforementioned factors. The proposed method seeks to maximize it while ensuring a balance between precision and feasibility. We recommend the choice of an RA_target_ value close to the best result in the experimental pool or best known value from a similar application.

To evaluate the impact of various factors on the area ratio, a linear regression model was developed. The choice of a linear regression model was based on its simplicity, interpretability, and ability to capture the linear relationships between predictor variables related to the scribing process (e.g., laser parameters) and the area ratio. By estimating the coefficients of the model, it was possible to identify the influence of each predictor variable on the area ratio. The results of the model were then analyzed to evaluate the scribing precision. After computing the RA values, as shown in Table 1, we observed that the last three conditions (conditions 147, 160, and 163) generated RA values of less than 20%. Since our purpose was to seek settings for large RA, we eliminated these last three rows for regression model building.

A first-order polynomial linear regression model was utilized to quantify the relationship between the laser scribing area ratio and the parameters involved, pulse energy (E), pulse duration (tp), and pulse frequency (fp). The polynomial equation is shown in Equation (8):

(8) $$\mathrm{Laser}\;\mathrm{Scribing}\;\mathrm{Area}\;(\mathrm{RA})=b_{0}+b_{1}E+b_{2}tp+b_{3}fp$$

where E is the pulse energy (µJ), tp is the pulse duration (ps), and fp is the pulse repetition rate (Hz). The coefficients $b_{0}$ through $b_{3}$ were estimated by the least-squares method to minimize the error between the predicted and actual laser scribing area ratios. The coefficient $b_{0}$ is the intercept, which represents the value of RA when all independent variables are set to zero; coefficients $b_{1},\;b_{2}$, and $b_{3}$ are the main effects. Note that coded variables were used to fit all the regression models in this study. A coded variable is generated by converting the physical reading of an independent variable into the range of −1 to +1. For example, the range of E (µJ) is from 24 to 360. Then, the low level (−1) corresponds to 24 while the high level (+1) corresponds to 360. The rest of the E (µJ) setting is linearly transformed. For example, E (µJ) = 60 is coded as (60 − (24 + range/2))/(range/2), where the range = 360–24.

Table 2 presents the estimated coefficient values along with their corresponding *p*-values. Smaller *p*-values indicate the greater significance of the variables contributing to the response. However, despite the significance of some variables, the R-squared value of the initial model was only 0.345. This suggests that approximately 34.5% of the variance in the response variable can be explained by variations in the laser parameters considered. To enhance the model’s predictive capability due to lack of fit, we decided to refit the model using a polynomial of degree = 2. The results of this refitted model are presented below:

(9) $$\mathrm{RA}=b_{0}+b_{1}E+b_{2}tp+b_{3}fp+b_{4}E\cdot tp+b_{5}E\cdot fp+b_{6}tp\cdot fp+b_{7}E^{2}+b_{8}{tp}^{2}+b_{9}{fp}^{2}\;b_{10}E\cdot tp\cdot fp$$

The regression models were reconfigured with a degree of 2 to augment their predictive capabilities. The degree = 2 model showcased a notable enhancement in the coefficient of determination (R-squared) in contrast to the degree = 1 model. The R-squared value surged from 34.5% to 83.2%, underscoring a significant advancement in the model’s adeptness at elucidating the variations within the response variable (RA). Roughly 83.2% of the observed fluctuations in RA could then be linked to the pertinent laser parameters. Despite this improvement, none of the *p*-values provided in Table 3 for the coefficients of individual predictor variables was small enough for any coefficient to be statistically significant. However, the *b*_0_ = 64.3156 suggests that the RA was close to the best experimentally observed value when the setting was set at the middle levels for all parameters. This is because regression model (9) was fitted via the coded variables for E, tp, and fp. In short, the maximal value for each variable was set to +1 and the minimal value was set to −1. Then, the setting 0 represented the center point. When all parameters were set to their center points, the estimated RA was at the *b*_0_ value.

### 4.3. MSE for Scribing Consistency Measurement

As shown in Figure 2, scribing cross sections vary even under the same scribing conditions. The possible reasons for such variations could be non-uniform laser intensity distribution over the writing track, which will lead to non-uniform material ejection and redeposition during laser scribing, and Al film thickness variations and surface contamination. To assess the consistency of the laser scribing, the mean square error (MSE) was used. After obtaining the cross-section profiles, the MSE of each laser scribing trial with the cubic spline model was calculated and provided a measure of the deviation of the laser scribing profile from the fitted curve, indicating how well the model could represent the actual profile. The MSE equation is listed as follows:

(10) $$MSE=\frac{1}{N}\sum {(Y}_{acutal}{-Y_{reference})}^{2}$$

In this equation, $Y_{acutal}$ represents the observed laser scribing value at a given location while $Y_{reference}$ represents the corresponding point on the estimated value from the cubic spline model, and N represents the total number of locations along the cross-section profile. Each blue profile in Figure 2 is an actual scribed cross-section profile, and its corresponding cubic spline model is shown in red. To compute the MSE, Equation (10) computed the squared difference between each point on the actual laser scribing trajectory and the corresponding point on the reference curve. These squared differences were then summed across all data points along a profile. Finally, the sum was divided by the total number of data points (N) to obtain the average squared deviation. MSE measured how much deviation of the laser scribing profile there was from the reference curve. A small MSE value indicated a more consistent and accurate reproduction of the scribing profile. Conversely, a large MSE value indicated a greater discrepancy between the actual and reference profiles. Monitoring the MSE values across different trials or experimental conditions can provide valuable insights into the precision and consistency of the laser scribing process, helping to identify areas for improvement and ensuring adherence to desired scribing specifications.

**Figure 4:**
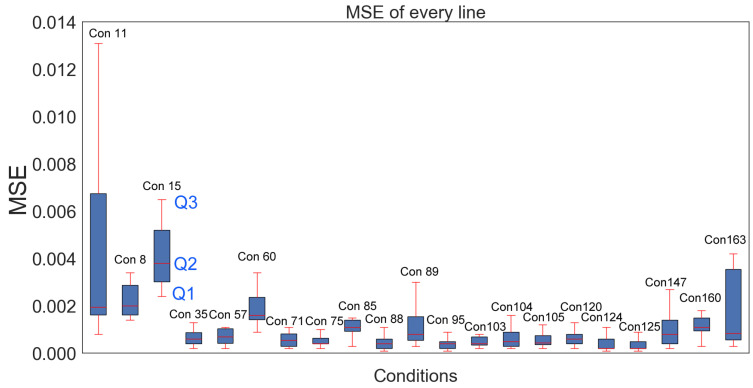
The distribution of MSE with quartile values Q1, Q2, and Q3 across 21 scribing conditions.

A small MSE value indicates a consistent scribe line and accurate reproduction of the scribe profile, while a large MSE suggests a significant deviation from the reference scribe line and indicates an issue with the scribing process. This information can help balance the choice of optimal operating settings. Figure 4 provides a comparison of the laser scribing in terms of MSE for selected scribing conditions. A boxplot displays the distribution of MSE values for each condition across 10 trials (i.e., 10 different scribes under the same conditions). A few scribing conditions, for example, conditions 11, 15, and 163 (depicted as Con 11, 15, and 163 in Figure 4), exhibited broad distributions of MSE values. This implies that their performances were not very consistent across 10 trials. Conversely, most scribing conditions had narrower MSE distributions, which suggests more consistent performances.

As shown in condition 15 in Figure 4, there are quartile values Q1, Q2, and Q3. Q1 (the first quartile) represents the 25th percentile of the MSE distribution, meaning that 25% of the laser scribes had MSE values below Q1. Q2 (the second quartile) represents the 50th percentile of the MSE distribution, which is equivalent to the median. It represents the middle value of the MSE distribution, with 50% of the laser scribes having MSE values below Q2 and 50% having MSE values above Q2. Q3 (the third quartile) represents the 75th percentile of the MSE distribution, meaning that 75% of the laser scribes had MSE values below Q3. The red lines above and below a box indicate the spread of the data points and can be used to identify outliers. In this case, we prefer small Q2 values since the smaller the Q2 values, the less deviation from the fitted profile. In addition, we also prefer small interquartile distances (i.e., Q3-Q1). If an interquartile range value is large, this may indicate that the distribution of MSE values is wide and that there is a large spread of values. This implies that the scribes under the same condition do not generate similar profiles. Conversely, if the interquartile range is small, this indicates that scribing profiles under the same conditions generate similar profiles more consistently. Additionally, if there are any laser scribes with MSE values that are significantly higher or lower than the rest of the values (i.e., long red lines outside the box), these could be identified as potential outliers, e.g., condition 11.

The range between Q1 and Q3 was relatively small for most conditions, indicating that the MSE values were somewhat consistent within each condition. Con 15 had the highest median MSE value (Q2), followed by Con 8 and Con 11. This suggests that these conditions may not generate repeatable scribes compared to the other conditions. Con 11 had the largest difference between Q3 and Q1, indicating that there was greater variability in MSE values for this condition. This may suggest that the laser scribing results were less consistent for this condition compared to the others.

A polynomial linear regression model was employed to establish the relationship between the mean square error (MSE) of the laser scribe profile and the laser parameters involved, including pulse energy, pulse duration, and pulse repetition rate. The polynomial equation was used to fit through the data points in the plot, allowing for the estimation of the MSE at different parameter values. Specifically, a polynomial of degree = 1 was used, which corresponded to a linear regression. By doing so, the initial relationship between the MSE and the laser parameters was examined. The polynomial equation took the form of

(11) $$\mathrm{MSE}=b_{0}+b_{1}E+b_{2}tp+b_{3}fb$$

The concept was analogous to the RA regression model, where the coefficients $b_{0}$ through $b_{3}$ were estimated through a least-squares method. This approach aimed to minimize the disparity between the predicted and actual MSE values. Table 4 exhibits the estimated coefficient values along with their corresponding *p*-values. Using this model, it was possible to analyze the effect of each parameter on the MSE, as well as any interactions between the parameters. The coefficient values *b*_1_, *b*_2_, and *b*_3_ of the main effect provided information about the magnitude and direction of the effect of each parameter on the MSE. A positive coefficient indicated that increasing the corresponding parameter would increase the MSE, while a negative coefficient indicated that increasing the parameter would decrease the MSE. By analyzing the coefficients, one could determine the optimal values of the independent variables to minimize the MSE.

The R-squared value of the model, which was 0.762, suggested that around 76.2% of the variance in the MSE could be accounted for by the variations in the laser parameters considered. To improve the model’s predictive capability, the model was refitted using a polynomial of degree = 2 and the corresponding results are presented below:

(12) $$\mathrm{MSE}=b_{0}+b_{1}E+b_{2}tp+b_{3}fb+b_{4}E\cdot tp+b_{5}E\cdot fb+b_{6}tp\cdot fp+b_{7}E^{2}+b_{8}{tp}^{2}+b_{9}{fp}^{2}+b_{10}E\cdot tp\cdot fp$$

The regression coefficients of the predictors and their corresponding *p* values are shown in Table 5. The refitted model provided a remarkable improvement in the predictive performance, as indicated by the R-squared value of 0.942. This substantial increase compared to the initial model (R-squared = 0.762) suggests that approximately 94.2% of the variability in the MSE can now be explained by the considered laser parameters. The enhanced R-squared value signifies that the quadratic polynomial provided a better fit to the data, effectively capturing a larger portion of the MSE variability. From the *p*-values from both Table 2 and Table 3, we observe that parameters E and fp are statistically significant.

The negative coefficient and significant *p*-value for E (−0.0185, *p* = 0.032) imply that increasing the pulse energy led to a reduction in MSE. Higher pulse energies may translate to more energy being deposited on the material surface during each laser pulse. This may result in a more rapid and precise material removal process, leading to lower MSE values. Similarly, the negative coefficient and significant *p*-value for fp (−0.0196, *p* = 0.025) indicate that increasing the normalized pulse repetition rate is associated with lower MSE values. Increasing the pulse repetition rate allowed less time for the material to cool between successive laser pulses, which could improve the material’s response to the laser ablation and lead to more consistent scribe profile quality. In addition, the coefficient for the interaction term E × fp was also significant and had a larger value than those of E and fp. This result suggests that if the parameter settings of E and fp were set at a high level, the coefficient of E × fp would further reduce MSE. Finally, the *p*-values of all other coefficients (including tp) showed no significant effect on the MSE as its coefficients had a *p*-value greater than 0.05. This result suggests that the interplay between pulse duration (tp) and pulse repetition rate (fp) does not significantly influence the MSE. It is possible that within the range of the data considered, the combination of pulse duration and repetition rate does not lead to significant changes in material removal dynamics or ablation mechanisms. Given this result, we shall focus on E and fp in searching for the optimal parameter setting for scribing accuracy and consistency.

### 4.4. Contour Plot Model for Exploring the Relationship between tp, E, RA, and MSE

**Figure 5:**
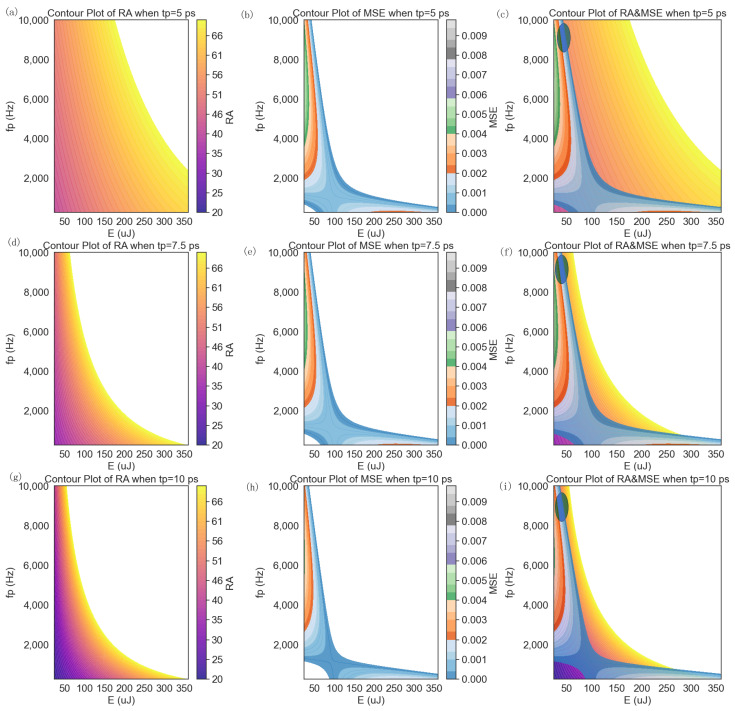
The contour plots of RA, MSE, and superimposed RA and MSE: (**a**–**c**) tp = 5 ps, (**d**–**f**) tp = 7.5 ps, (**g**–**i**) tp = 10 ps (**c**,**f**,**i** are superimposed charts from their previous two charts).

In our comprehensive exploration of laser scribing quality through cross-section scribing profiles, various analytical techniques were employed to gain insights into the relationship between pulse energy (E), pulse duration (tp), and the quality metrics of interest [32]. One of the key tools utilized in this investigation was the contour plot analysis. The contour plot analysis played a crucial role in visually representing response cross sections (either MSE or RA) between two variables, E and tp. Specifically, a contour plot illustrated the relationship between the E (*x*-axis) and the fp (*y*-axis) in terms of two performance metrics: RA and MSE [33], as shown in Figure 5, where Figure 5a–c are under the condition tp = 5 ps, Figure 5d–f are under the condition tp = 7.5 ps, and, finally, Figure 5g–i are under the condition tp = 10 ps. The colored areas are feasible regions. The white areas are infeasible because the RA should be smaller than 100% and the MSE should be positive. Parameters fp and E were chosen since both regression Equations (11) and (12) indicated that E, fp, and E-fp interactions were statistically significant.

We favor regions with high RA values in Figure 5a. The choice of tp = 5 ps was primarily guided by the analysis of the 2nd order regression model on RA, where the center point (i.e., E, tp, and fp were set at their middle levels) yielded favorable RA values. This specific setting, tp = 5 ps, served as the central point for tp. Upon closer examination of Figure 5b, two areas shaded in dark blue reveal low MSE values.

In the context of multiple objective optimizations [34], it is important to note that the optimal solution for one response may not align with the optimal solution for another response. Hence, a balanced and compromised optimal solution must be achieved. In Figure 5c, we overlay two contour plots from 5a and 5b. The central points for E and fp were set at 168 uJ and 4875 Hz, respectively. Notably, this configuration was derived from Equation (9) and lay beyond the feasible MSE region. The range of optimal parameters is highlighted by the blue shaded circle, and several compromised parameter configurations are selected and detailed in Table 6. Employing a similar process, potential compromised solutions were generated for tp = 7.5 ps from Figure 5d–f and for tp = 10 ps from Figure 5g–i. All potential compromised optimal solutions are summarized in Table 6.

**Figure 6:**
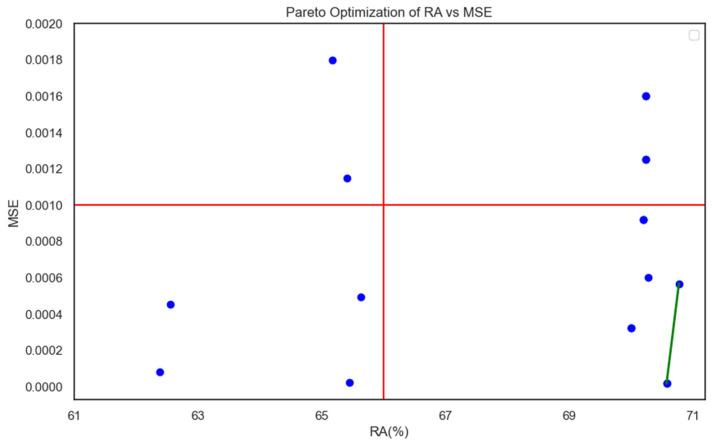
A scatter plot depicting (RA, MSE) values across 18 parameter settings (the green line is the Pareto front, the two (RA, MSE) pairs dominate all the other pairs). The blue dots represent the 18 distinct parameter settings (S1 through S18) shown in Table 6. The green line represents the Pareto front, where all compromised solutions are dominated by solutions on this front.

Figure 6 shows a scatter plot for all 18 parameter settings based on the RA and MSE values listed in Table 6 in an attempt to search for a Pareto optimal setting [35]. A Pareto solution dominates in both RA and MSE. We do not have a Pareto solution in this case. Since we prefer a large RA and small MSE, the lower right-hand corner in Figure 6 is the preferred region, consisting of machine settings S9, S10, S16, S17, and S18. Specifically, S18 had the lowest MSE while RA = 70.58%, while S16 had the largest RA = 70.78% but its MSE was larger than that of S18. Between solutions S16 and S18, a Pareto front could be generated. All the other solutions were dominated by this front. In other words, no other solutions could outperform S16 or S18 in terms of either RA or MSE. A decision maker or process engineer can now focus on choosing the best compromised solution between S16 and S18 or another combination on the Pareto front.

## 5. Conclusions and Future Work

In conclusion, this study explored the laser scribing quality of a thin aluminum film coated on a silicon substrate through cross-section scribing profiles and identified potential optimal operating regions using contour plots. We propose the use of cross-section profiles for the more precise evaluation of scribing quality, rather than just scribing width and depth. A cubic spline model, fitting the cross-section profile, served as the base for measuring scribing quality via the proposed statistics RA and MSE. Both scribing accuracy and consistency were considered via contour plots. A Pareto optimization procedure was used to search for a compromised solution.

The findings from this study provide valuable guidance for parameter optimization in laser scribing processes. By focusing on the identified optimal operating points, practitioners can achieve stable and satisfactory scribing quality while considering the critical roles of pulse energy and pulse duration. Future work is required to further explore the underlying mechanisms that contribute to the observed optimal regions and investigate additional factors that may influence laser scribing quality, such as laser power and beam spot size. Additionally, advanced modeling techniques can be employed to develop predictive models that encompass a wider range of parameters and accurately estimate scribing quality based on the identified optimal operating points. This research lays the foundation for further advancements in laser scribing processes and opens new possibilities for improved efficiency and precision in various applications.

## Figures and Tables

**Table 1:** Computing the RA and MSE values.

Condition	E (µJ)	tp (ps)	fp (Hz)	MSE	RA
Con 8	240	10	500	0.00361	37.94
Con 11	240	1	250	0.00638	44.61
Con 15	360	2	250	0.00447	49.86
Con 35	120	10	1000	0.00075	46.43
Con 57	24	0.5	10,000	0.00107	42.06
Con 60	24	10	10,000	0.00192	69.94
Con 71	48	0.184	2000	0.00059	40.9
Con 75	48	10	2000	0.00055	32.18
Con 85	105	10	1000	0.00111	29.2
Con 88	30	1	2000	0.00047	41.08
Con 89	30	2	2000	0.00129	47.9
Con 95	30	10	5000	0.00042	53.43
Con 103	60	0.5	1000	0.00068	41.24
Con 104	60	1	1000	0.00076	43.4
Con 105	60	2	1000	0.00061	44.17
Con 120	60	10	2000	0.00081	44
Con 124	60	2	3000	0.00043	43.5
Con 125	60	10	3000	0.00038	42.85
Con 147	15	0.5	50,000	0.00107	11.66
Con 160	10	10	100,000	0.00114	19.35
Con 163	12	1	100,000	0.00184	10.95

**Table 2:** The estimated coefficients and associated *p*-values for each predictor variable in a linear model (degree = 1) of laser scribing area ratio.

Variable	Coefficient (*b_i_*)	*p*-Value
Intercept	51.454	0.000
E	4.017	0.309
tp	−0.078	0.970
fp	9.500	0.018

**Table 3:** The estimated coefficients and associated *p*-values for each predictor variable in a linear model (degree = 2) of laser scribing area ratio.

Variable	Coefficient (*b_i_*)	*p*-Value
Intercept	64.3156	0.258
E	7.8994	0.900
tp	71.7138	0.300
fp	14.4419	0.817
E·tp	69.076	0.335
E·fp	2.3735	0.970
tp·fp	77.5716	0.285
$E^{2}$	−0.5282	0.950
${tp}^{2}$	−10.109	0.264
${fp}^{2}$	−2.7738	0.752
E·fp·tp	65.036	0.347

**Table 4:** The estimated coefficients and associated *p*-values for each predictor variable in a linear model (degree=1) of MSE.

Variable	Coefficient (*b_i_*)	*p*-Value
Intercept	0.0035	0.000
E	0.0029	0.000
tp	−0.0002	0.333
fp	0.0007	0.113

**Table 5:** The estimated coefficients and associated *p*-values for each predictor variable in a linear model (degree = 2) of MSE.

Variable	Coefficient (*b_i_*)	*p*-Value
Intercept	−0.0128	0.069
E	−0.0185	0.032
tp	0.0012	0.873
fp	−0.0196	0.025
E·tp	0.0012	0.884
E·fp	−0.0209	0.019
tp·fp	0.0019	0.807
$E^{2}$	−0.0036	0.007
${tp}^{2}$	−0.0005	0.581
${fp}^{2}$	−0.0016	0.150
E·fp·tp	0.0016	0.830

**Table 6:** The potential optimal parameter settings from contour plots.

Setting	tp (ps)	E (μJ)	fp (Hz)	RA	MSE
S1	5	31	10,000	65.64	4.9311 × 10^−4^
S2	5	34	9705	65.45	2.1304 × 10^−5^
S3	5	27	10,000	65.41	1.148 × 10^−3^
S4	5	24	10,000	65.18	1.8 × 10^−3^
S5	5	38	7833	62.56	4.5348 × 10^−4^
S6	5	41	7636	62.39	7.94 × 10^−5^
S7	7.5	24	10,000	70.25	1.6 × 10^−3^
S8	7.5	27	9311	70.24	1.254 × 10^−3^
S9	7.5	31	8720	70.21	9.21 × 10^−4^
S10	7.5	34	8227	70.28	6.01 × 10^−4^
S11	7.5	38	7735	70.01	3.23 × 10^−4^
S12	7.5	41	7439	70.58	1.65 × 10^−5^
S13	10	24	10,000	70.25	0.16 × 10^−3^
S14	10	27	9311	70.24	0.1254 × 10^−3^
S15	10	31	8720	70.21	0.9209 × 10^−4^
S16	10	34	8326	70.78	5.675 × 10^−4^
S17	10	38	7735	70.01	3.234 × 10^−4^
S18	10	41	7439	70.58	1.6529 × 10^−5^

## Data Availability

Data underlying the results presented in this paper are not publicly available at this time but may be obtained from the authors upon reasonable request.
